# Profiles for identifying problematic dietary habits in a sample of recreational Spanish cyclists and triathletes

**DOI:** 10.1038/s41598-021-94660-0

**Published:** 2021-07-26

**Authors:** José J. Muros, Emily Knox, Daniel Hinojosa-Nogueira, José Á. Rufián-Henares, Mikel Zabala

**Affiliations:** 1 Department of Didactics of Corporal Expression, University of Granada, 18071 Granada, Spain; 2 Andalusian School of Public Health (EASP), 18071 Granada, Spain; 3 Department of Nutrition and Food Science, Institute of Nutrition and Food Technology, Biomedical Research Centre, University of Granada, 18071 Granada, Spain; 4 Department of Physical Education and Sport, University of Granada, 18071 Granada, Spain

**Keywords:** Nutrition, Public health

## Abstract

There is a lack of sufficient information on the dietary intake and nutritional supplementation of recreational endurance athletes throughout the year. The present observational study sought to assess the dietary intake and nutritional supplementation habits of recreational cyclists and triathletes from Spain. 4,037 cyclists and triathletes completed self-report measures. Nutritional profiles were developed and differences were examined according to sporting discipline and gender. Differences between groups were compared using the Mann–Whitney U or chi-squared test. Next, micro- and macro-nutrients were grouped according to whether or not guideline intake amounts were met. The clustering of dietary habits was then examined via K-means cluster analysis. Triathletes took more supplements than cyclists (X2 = 36.489; p value = .000) and females took more supplements than males (X2 = 5.920; p value = .017). Females and triathletes reported greater protein and CHO consumption than males and cyclists, respectively. Triathletes also reported a higher consumption of total fat, MUFA, PUFA, EPA, DHA and fibre. Females and triathletes tended to consume more vitamins and minerals than males and cyclists, respectively. Two main dietary habit clusters emerged which may be used to inform nutritional interventions targeting recreational athletes not meeting nutritional requirements. There is an imbalance in the main nutrients making up the diet of recreational Spanish athletes, characterised by insufficient CHO and excessive protein.

## Introduction

Endurance sports such as cycling and triathlon are becoming increasingly popular. In recent years, a number of events have been organised to encourage people to take up endurance sports, with events lasting between 30 min and 2 h being increasingly popular amongst recreational athletes^1^.

Many individuals engage in recreational sport, with 103,631 members of federated cycling and triathlon clubs being registered in Spain. Despite this, few studies have been conducted on eating habits and nutrient intake within this group. For all individuals, the diet should provide sufficient energy from carbohydrate (CHO), protein and fat, alongside appropriate quantities of vitamins and minerals. Whilst the diet of non-athletes and athletes should be proportionally similar, physically active individuals must consume a greater quantity of macronutrients in order to meet their greater energy demands. However, studies have demonstrated that endurance athletes do not meet the proposed goal of 5 to 7 g of CHO per kg of body weight (BW) a day^2^. On the other hand, protein intake has been shown to exceed recommended daily intakes for endurance athletes^3^, whilst fat consumption has also been shown to be excessive^4^. Insufficient CHO intake prevents muscle glycogen replacement and leads to an imbalanced blood glucose level during exercise^5^. Further, athletes with high protein diets could also suffer from metabolic acidosis^6^. Both of these outcomes can adversely affect exercise performance. High protein intake is related with urea production and oxidation of the carbon skeleton, whilst also interfering with appropriate CHO intake^7^. It might be appropriate to reduce fat intake in order to make room to increase CHO intake^8^. Increased fat intake accompanied by insufficient physical activity is associated with a higher risk of cardiovascular disease and obesity. However, when undertaking daily intensive physical aerobic training, a higher consumption of fat is not linked with higher fat mass or blood cholesterol in athletes. On the other hand, it is linked with an increase in homocysteine levels in the body, with this being related with cardiovascular diseases^9^.

In contrast to macronutrients, athletes or physically active individuals do not have special RDA recommendations for vitamins and minerals relative to the general population. Further, as physically active people are likely to consume more macronutrients in their diet, evidence suggests that additional micronutrient needs are likely to be met^10^. Despite this, some individuals may use nutritional supplements to address some of these needs. Some authors have argued that nutritional supplements are unnecessary when a well-balanced diet is followed^11^. Nonetheless, dietary supplement use has grown significantly over recent years due to aggressive marketing campaigns online and on social media, and the efforts of nutritional supplement companies to sponsor outstanding athletes^12^. However, there are significant risks associated with the use of dietary supplements, such as the absence of active ingredients, presence of harmful substances and even the presence of doping agents^13,14^. Further, an excessive or deficient intake of certain vitamins and minerals can lead to serious alterations in the body at both a metabolic and psychological level. Despite these potential issues to both health and performance, the supply of dietary supplements is not regulated and no type of medical referral is required for their acquisition. As a result, many recreational athletes decide to take such supplements without expert guidance and consume quantities that exceed recommended daily intake levels. This can lead to issues, for instance, high dosages of antioxidant supplements have negative effects on exercise-induced adaptation processes^15^.

Cycling and triathlon are endurance sports, however, triathlon is a “new” sport relative to cycling. Cyclists are often led to follow “old” nutritional strategies passed down through word of mouth. In contrast, triathletes do not have this problem, with triathlon being considered to be a “new” sport relative to cycling. Additionally, female Spanish triathletes have been reported to have a higher socioeconomic status than cyclists^16^ and this could influence their nutritional habits. We therefore hypothesised that nutritional habits would differ between participants engaged in cycling and those engaged in triathlon. Although the dietary intake and nutritional supplementation of elite and non-elite cyclists and triathletes during training has been documented previously, there is a lack of information on the dietary intake and nutritional supplementation followed by recreational Spanish endurance athletes in one full year. The main aim of the present observational study was to assess the dietary intake of recreational cyclists and triathletes from Spain by developing nutritional profiles to identify unhealthy dietary habits, particularly those involving supplementation, and, in this way, highlight targets for intervention. Differences were also examined according to sporting discipline and gender.

## Methods

### Design

The study was cross-sectional and counted on a sample of 4,037 recreational cyclists and triathletes (male: 90.1%) from across Spain. A total of 337 participants were excluded for failing to complete some element of testing or reporting extreme total energy intake values (<800 kcal > 4000 kcal for men and <500 kcal > 3500 kcal for women), as recommended for analysis in nutritional epidemiology^17^. A demographic self-report form and food frequency questionnaire (FFQ) were entered into the application Google Drive^®^ (Alphabet, Mountain View, USA). Ethical principles of the Declaration of Helsinki for medical research were adhered to. Ethical approval was granted by the Ethics Committee of the University of Granada, Spain (N º 883).

### Demographic form

The final questionnaire and instructions for its completion were emailed to the Royal Spanish Cycling Federation and the Spanish Triathlon Federation, who forwarded to all associated members. To be eligible for inclusion, participants had to be over eighteen years old and have previously given permission to their respective federation to contact them via e-mail. Participants provided informed consent before completing the questionnaire. The research was conducted during 2016. Some 75,871 (male: 95%) cyclists and 27,760 (male: 82.3%) triathletes, respectively, were federated in Spain during 2016. Of these, 1,861 (male: 95.8%) and 1,839 (male: 84.3%), respectively, satisfactorily completed the questionnaire. All questionnaires were completed at the same time of year.

Participants were asked to self-report their sex, date of birth, years practicing their sport, weekly training hours, height, weight and sport discipline (cycling, triathlon).

### Dietary assessment

The 136-item FFQ was used to assess dietary intake. This has demonstrated high validity and reliability within similar populations^18^. Commonly used portion sizes were specified (cup, teaspoon, etc.) for each food item. Participants were asked to report their average consumption of a specified unit of foodstuff over the previous year. Nine options were offered: never or hardly ever, one to three times a month, once a week, two to four times a week, five to six times a week, once a day, two to three times a day, four to six times a day and more than six times a day. The chosen response was converted into a daily intake (for example responses of 5–6 times a week were converted to 0.78 servings per day). A seasonal variation factor was considered for foodstuffs whose consumption was not regular throughout the year. The questionnaire also included the following questions: “If during the past year you took sport supplements or dietary products (bran, evening primrose oil, milk with omega-3 fatty acids, flavonoids, etc.), please indicate the BRAND”; “Please indicate the FREQUENCY with which you took them”. i-Diet software (GNS, Spain) was used to estimate energy and daily nutrient intake^19^. Data for the nutritional content of dietary supplements was obtained from product labels and added to daily intake calculations by a trained nutritionist. Participants were considered to be dietary supplements consumers when they reported consuming these products at least one time per week during a year.

We defined 19 food groups (milk and dairy products, eggs, meat and meat products, fish and seafood, vegetables, pulses, fruits, nuts, pasta/potatoes/rice, refined cereals and their derivatives, wholegrain cereals and their derivatives, olive oil, other oils and fats, baked goods and pastries, sugar and sweets, beer/wine, alcoholic beverages, soft drinks, and coffee/tea) according to their nutritional similarities.

We classified unprocessed or minimally processed foods as: edible parts of plants (seeds, fruits, leaves, stem and roots) animals (muscle, offal, eggs and milk) and fungi or algae. Ultra-processed foods were classified as: soft drinks, sweet or savoury packaged snacks, reconstituted meat products and pre-prepared frozen dishes. Classifications were made following the groupings proposed by Monteiro et al.^20^.

### Statistical analysis

Means are presented for all quantitative variables alongside standard deviations. Data normality was tested using the Kolmogorov–Smirnov test with Lilliefors correction and homoscedasticity was assessed using the Levene test. Following verification that data were non-normally distributed, Mann–Whitney U tests were employed for two-group comparisons. Qualitative variables are presented according to their frequency distribution. Associations between qualitative variables were determined using chi-squared analysis with Bonferroni correction.

In order to establish profiles, dietary patterns for all macronutrients, vitamins and minerals were individually categorised as being insufficient, adequate or excessive. Vitamin or mineral consumption was considered to be excessive when intake values were reported to be higher than tolerable upper intake levels (UL) for that micronutrient. Consumption was deemed deficient when intake of any vitamin or mineral was lower than the recommended dietary allowance (RDA). Adequate intake (AI) was used in cases where RDA was not relevant. Macronutrient, cholesterol and fibre intake was considered excessive or inadequate when reported values were higher or lower, respectively, than that recommended by the Spanish Society of Community Nutrition ([SENC] 50–60% CHO, 30–35% lipids, 10–15% protein, less than 300 mg/day cholesterol and less than 25 g/day fibre). Poor lipid quality was established when (PUFA + MUFA)/SFA ≥ 2.

Once consumption of each micro- and macro-nutrient was categorised, we proceeded to examine the way in which dietary habits clustered together via K-means cluster analysis. The large sample size meant that hierarchical cluster analysis was not appropriate. In order to standardise variables for use in K-means analysis, multiple correspondence analysis was first conducted to establish principal components which were then entered into K-means clustering. Clusters of 2, 3, 4 and 5 were tested. The maximum number of iterations was set at 10 and the convergence factor was set at 0. The most appropriate cluster model was selected based on the number of iterations needed for convergence, variable contribution to the cluster (F-test) and examination of a bar graph of cluster mean centres.

Data were analysed using AMOS and the IBM-SPSS version 25.0 statistical programme for Windows (Armonk, NY: IBM Corp). The level of significance was set at 0.05.

## Results

Characteristics of the sample regarding age, experience, training hours, weight, height and BMI are presented in Table 1.

**Table 1:** Participant general characteristics.

Characteristic	Mean ± SD	Characteristic	Mean ± SD
**Age (years)**		**Age (years)**	
Women	32.15 ± 9.35	Triathletes	34.66 ± 8.54
Men	36.76 ± 9.10	Cyclists	37.93 ± 9.59
**Experience (years)**		**Experience (years)**	
Women	6.22 ± 7.32	Triathletes	5.56 ± 6.30
Men	9.75 ± 9.38	Cyclists	13.19 ± 10.11
**Training (hours per week)**		**Training (hours per week)**	
Women	11.02 ± 5.04	Triathletes	11.57 ± 4.70
Men	11.23 ± 4.56	Cyclists	10.85 ± 4.49
**Height (cm)**		**Height (cm)**	
Women	165.03 ± 6.08	Triathletes	175.33 ± 7.74
Men	176.7 ± 6.40	Cyclists	175.80 ± 6.77
**Weight (kg)**		**Weight (kg)**	
Women	57.59 ± 7.22	Triathletes	70.54 ± 9.95
Men	73.60 ± 8.99	Cyclists	73.45 ± 9.92
**BMI (kg/m**^**2**^**)**		**BMI (kg/m**^**2**^**)**	
Women	21.12 ± 2.13	Triathletes	22.86 ± 2.24
Men	23.54 ± 2.39	Cyclists	23.73 ± 2.62

SD: Standard deviation; BMI: Body mass index.

Females consumed greater amounts of vegetables, fruits, nuts, wholegrain cereals, olive oil and coffee or tea (p < 0.05). Males presented higher intakes of eggs, meat and meat products, pulses, pasta, potatoes and rice, refined cereals, baked goods and pastries, sugar and sweets, soft drinks and alcoholic beverages including beer and wine (p < 0.05). Triathletes consumed greater levels of fish and seafood, vegetables, fruits, nuts, wholegrain cereals, olive oil, and coffee or tea. Cyclists presented a higher intake of milk and dairy products, pulses, refined cereals, oils and fats other than olive oil, soft drinks and alcoholic beverages including beer and wine (p < 0.05) (Table 2). In addition, females and triathletes showed a higher intake of unprocessed foods and a lower intake of ultra-processed foods than males and cyclists (p < 0.05).

**Table 2:** Consumption of each food group according to sex and sport speciality.

	Overall (N = 3700 )	Male (n = 3333 )	Female (n = 367 )	p value	Triathlete (n = 1839)	Cyclist (n = 1861)	p value
**Food groups (serving/day)**							
Milk and dairy products	2.39 ± 1.73	2.42 ± 1.73	2.26 ± 1.75	.099	2.30 ± 1.61	2.49 ± 1.85	.001*
Eggs	0.37 ± 0.29	0.37 ± 0.29	0.34 ± 0.34	.000**	0.38 ± 0.31	0.36 ± 0.27	.069
Meat and meat products	1.53 ± 1.03	1.55 ± 0.98	1.34 ± 1.31	.000**	1.50 ± 0.97	1.57 ± 1.08	.296
Fish and seafood	0.83 ± 0.62	0.83 ± 0.62	0.84 ± 0.55	.204	0.88 ± 0.66	0.78 ± 0.57	.000**
Vegetables	2.27 ± 1.64	2.19 ± 1.56	3.03 ± 1.99	.000**	2.32 ± 1.61	2.23 ± 1.67	.036*
Pulses	0.35 ± 0.26	0.35 ± 0.26	0.32 ± 0.25	.002*	0.34 ± 0.26	0.36 ± 0.26	.000**
Fruits	3.09 ± 2.26	3.03 ± 2.21	3.53 ± 2.60	.000**	3.20 ± 2.28	2.96 ± 2.22	.000**
Nuts	0.53 ± 0.71	0.52 ± 0.70	0.62 ± 0.75	.025*	0.62 ± 0.81	0.45 ± 0.58	.000**
Pasta, potatoes, rice	0.82 ± 0.51	0.83 ± 0.52	0.71 ± 0.42	.000**	0.83 ± 0.54	0.81 ± 0.48	.533
Refined cereals	0.96 ± 1.13	0.98 ± 1.14	0.74 ± 0.42	.000**	0.94 ± 1.13	0.98 ± 1.11	.049*
Whole cereals	0.72 ± 0.93	0.69 ± 0.92	0.91 ± 0.96	.000**	0.79 ± 0.94	0.64 ± 0.91	.000**
Olive oil	1.41 ± 1.23	1.36 ± 1.16	1.88 ± 1.62	.000**	1.47 ± 1.25	1.35 ± 1.20	.000**
Other oils and fats	0.22 ± 0.46	0.22 ± 0.45	0.24 ± 0.51	.607	0.21 ± 0.49	0.23 ± 0.42	.001*
Baked goods and pastries	0.73 ± 0.96	0.75 ± 0.99	0.60 ± 0.60	.015*	0.72 ± 0.93	0.74 ± 0.98	.301
Sugar and sweets	1.62 ± 1.44	1.63 ± 1.45	1.45 ± 1.29	.007*	1.59 ± 1.45	1.64 ± 1.42	.233
Beer/wine	0.51 ± 0.76	0.54 ± 0.78	0.28 ± 0.41	.000**	0.43 ± 0.61	0.59 ± 0.87	.000**
Alcoholic beverage	0.03 ± 0.08	0.03 ± 0.09	0.02 ± 0.04	.000**	0.03 ± 0.07	0.03 ± 0.10	.022*
Soft drinks	0.37 ± 0.69	0.38 ± 0.71	0.24 ± 0.50	.000**	0.31 ± 0.60	0.43 ± 0.76	.000**
Coffee/tea	1.39 ± 1.30	1.38 ± 1.31	1.42 ± 1.26	.212	1.43 ± 1.31	1.34 ± 1.30	.006*
Unprocessed food (g/d)	1295.4 ± 610.6	1283.8 ± 601.4	1400.5 ± 680.7	.000**	1307.1 ± 603.1	1283.9 ± 617.7	.047*
Ultra-processed food (g/d)	173.6 ± 180.0	177.6 ± 183.5	137.3 ± 139.7	.000**	159.8 ± 157.9	187.1 ± 198.5	.000**

g/d: grams per day. *p < .05, **p < .001.

Daily intake of energy and macronutrients according to sex and sport discipline is shown in Table 3. Males reported higher energy intake and lower ingestion of protein and CHO in terms of g/kg of body weight (BW) than females. Males also reported lower fat percentages, in addition to having lower MUFA, PUFA and cholesterol intake than females. Triathletes reported a higher intake of protein and CHO in terms of g/kg of BW than cyclists. Triathletes also reported higher total fat, MUFA, PUFA, EPA, DHA and fibre consumption than cyclists.

**Table 3:** Average daily energy and macronutrient intake with differences indicated between males and females and between triathletes and cyclists.

	Overall (N = 3700)	RI/PRIs^†^/AI^‡^	Male (n = 3333)	Female (n = 367)	p value	Triathlete (n = 1839 )	Cyclist (n = 1861)	p value
**Nutrient**								
Energy (kcal)	2083.4 ± 610.0		2091.3 ± 614.3	2011.7 ± 564.4	.028*	2094.3 ± 612.2	2072.6 ± 607.7	.208
Protein (g)	99.5 ± 30.6		100.0 ± 30.9	95.6 ± 27.4	.035*	101.1 ± 31.2	98.0 ± 30.0	.002*
Protein (%)	19.4 ± 4.1		19.4 ± 4.1	19.4 ± 4.2	.863	19.7 ± 4.3	19.2 ± 3.9	.002*
Protein (g/kg)	1.4 ± 0.5	0.83^†^	1.4 ± 0.5	1.7 ± 0.5	.000**	1.5 ± 0.5	1.4 ± 0.5	.000**
CHO (g)	246.0 ± 88.1		247.1 ± 88.6	235.8 ± 82.0	.036*	246.2 ± 88.8	245.7 ± 87.2	.871
CHO (%)	46.8 ± 7.1	45–60	46.9 ± 7.1	46.5 ± 7.3	.558	46.6 ± 7.1	47.1 ± 7.1	.060
CHO (g/kg)	3.5 ± 1.4		3.4 ± 1.4	4.2 ± 1.6	.000**	3.6 ± 1.4	3.4 ± 1.4	.002*
Fibre (g)	33.4 ± 15.0	25^‡^	33.0 ± 14.9	36.8 ± 15.2	.000**	34.5 ± 15.3	32.3 ± 14.6	.000**
Fat (g)	72.2 ± 25.2		72.1 ± 25.3	72.7 ± 25.0	.699	73.2 ± 25.1	71.1 ± 25.3	.005*
Fat (%)	31.2 ± 6.2	20–35	31.1 ± 6.2	32.5 ± 6.5	.000**	31.5 ± 6.1	30.9 ± 6.3	.001*
SFA (g)	23.2 ± 7.8		25.0 ± 7.9	21.9 ± 7.0	.082	23.3 ± 7.8	23.1 ± 7.9	.334
SFA (%)	10.0 ± 2.5		10.8 ± 2.4	9.8 ± 2.5	.366	10.0 ± 2.4	10.0 ± 2.5	.919
MUFA (g)	34.3 ± 11.2		34.2 ± 11.1	35.8 ± 11.5	.004*	34.9 ± 11.1	33.8 ± 11.3	.004*
MUFA (%)	14.8 ± 3.5		14.7 ± 3.5	16.0 ± 3.9	.000**	15.0 ± 3.5	14.7 ± 3.6	.005*
PUFA (g)	14.7 ± 5.3		14.6 ± 5.3	15.0 ± 5.5	.101	15.0 ± 5.4	14.2 ± 5.3	.000**
PUFA (%)	6.4 ± 1.8		6.3 ± 1.7	6.7 ± 2.0	.000**	6.4 ± 1.8	6.2 ± 1.7	.000**
EPA (mg)	129.5 ± 133.6		130.0 ± 132.8	125.2 ± 140.6	.300	136.0 ± 139.9	123.2 ± 126.8	.000**
DHA (mg)	376.3 ± 257.5		377.1 ± 259.0	368.9 ± 244.1	.707	393.3 ± 261.1	359.4 ± 252.9	.000**
Cholesterol (mg)	326.8 ± 119.9		330.1 ± 119.4	297.0 ± 120.8	.000**	329.0 ± 132.1	324.7 ± 116.6	.410

CHO: Carbohydrates; SFA: Saturated fatty acid; MUFA: Monounsaturated fatty acid; PUFA: Polyunsaturated fatty acid; EPA: Eicosapentaenoic acid; DHA: Docosahexaenoic acid.; RI: Reference intake; PRIs: Population reference intake; AI: Adequate intake *p < .05, **p < .001.

Table 4 shows average daily vitamin and mineral intake according to sex and sport discipline. Males reported lower vitamin A, B1, B3, B6, B9, C, D and E intakes, and lower intakes of the minerals iron, sodium, potassium and magnesium than females. Males reporter higher ingestions of vitamin B12 than females. Triathletes reported a higher consumption of vitamins A, B1, B2, B3, B6, B9, C, D and E than cyclists, as well as a higher ingestion of the minerals iron, zinc, magnesium and iodine.

**Table 4:** Average daily vitamin and mineral intake according to sex and discipline.

	Overall (N = 3700 )	Male (n = 3333 )	Female (n = 367 )	p value	Triathlete (n = 1839)	Cyclist (n = 1861)	p value
**Vitamins**							
A (µg)	2228.0 ± 1707.6	2182.0 ± 1686.9	2646.3 ± 1835.1	.000**	2320.1 ± 1773.5	2137.1 ± 1635.4	.001
B1 (mg)	1.8 ± 1.1	1.8 ± 1.1	1.9 ± 0.8	.000**	1.9 ± 1.2	1.8 ± 1.0	.000**
B2 (mg)	3.2 ± 7.5	3.2 ± 7.4	3.4 ± 8.1	.087	3.4 ± 8.1	3.0 ± 6.8	.001*
B3 (mg)	29.2 ± 13.7	29.1 ± 13.8	29.8 ± 12.7	.036*	30.4 ± 14.2	27.9 ± 13.1	.000**
B6 (mg)	2.9 ± 1.4	2.9 ± 1.4	3.1 ± 1.5	.008*	3.1 ± 1.6	2.8 ± 1.3	.000**
B9 (µg)	464.2 ± 192.3	461.0 ± 192.3	493.0 ± 190.4	.000**	475.7 ± 199.5	452.8 ± 184.3	.000**
B12 (µg)	8.1 ± 17.7	8.2 ± 18.6	7.1 ± 4.5	.002*	8.5 ± 24.1	7.7 ± 7.1	.119
C (mg)	286.8 ± 169.7	282.1 ± 167.6	329.8 ± 181.8	.000**	297.2 ± 175.5	276.6 ± 163.1	.000**
D (µg)	4.7 ± 3.1	4.7 ± 3.1	4.9 ± 2.9	.053	5.1 ± 3.4	4.3 ± 2.7	.000**
E(mg)	12.6 ± 7.4	12.4 ± 7.3	14.1 ± 8.1	.000**	13.3 ± 7.9	11.8 ± 6.7	.000**
**Minerals**							
Calcium (mg)	1044.9 ± 399.0	1044.1 ± 403.5	1052.2 ± 356.2	.234	1046.7 ± 399.2	1043.2 ± 398.9	.787
Iron (mg)	20.1 ± 122.1	19.8 ± 11.7	22.3 ± 15.4	.000**	21.0 ± 14.0	19.2 ± 9.9	.000**
Sodium (mg)	4469.9 ± 2347.2	4430.9 ± 2311.6	4823.8 ± 2625.8	.010*	4463.6 ± 2351.7	4476.1 ± 2343.3	.987
Potassium (mg)	4755.1 ± 1737.2	4738.3 ± 1744.7	4907.4 ± 1661.9	.017*	4754.9 ± 1713.4	4755.3 ± 1760.8	.673
Zinc (mg)	12.2 ± 3.7	12.2 ± 3.8	12.1 ± 3.5	.861	12.4 ± 3.8	12.0 ± 3.6	.000**
Magnesium (mg)	477.9 ± 183.5	476.3 ± 185.3	492.7 ± 165.0	.005*	488.4 ± 186.9	467.6 ± 179.5	.000**
Iodine (µg)	155.8 ± 78.2	155.5 ± 77.7	158.8 ± 83.1	.523	161.5 ± 80.6	150.2 ± 75.4	.000**

*p < .05, **p < .001.

With regards to sport discipline, triathletes reported a higher intake of dietary supplements than cyclists (X^2^ = 36.489; p value = 0.000). Male triathletes reported a higher intake of dietary supplements than male cyclists (X^2^ = 32.264; p value = 0.000), with no differences emerging between female triathletes and female cyclists (X^2^ = 0.719; p value = 0.429). With regards to sex, females reported a higher intake of dietary supplements than males (X^2^ = 5.920; p value = 0.017). However, no differences were found between male and female triathletes (X^2^ = 0.638; p value = 0.420), or between male and female cyclists (X^2^ = 1.566; p value = 0.235).

**Figure 1:**
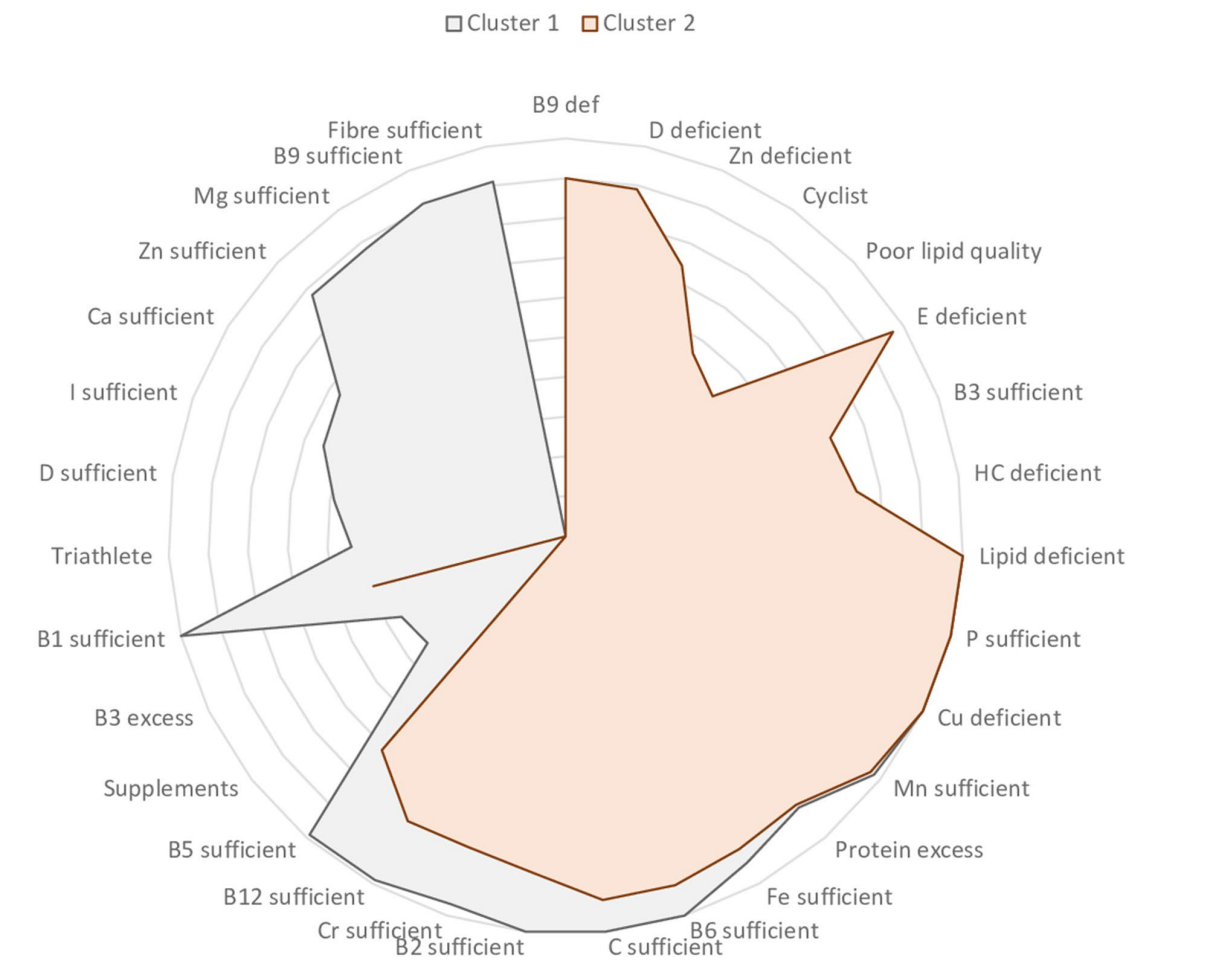
Graph of mean centres for all micro- and macronutrients and cluster formation. Footnote: Cluster 1 (54.4%) – somewhat deficient: carbohydrate deficient: lipid deficient, excess vitamin B3, vitamin E deficient, excess protein, takes supplements, triathlete; Cluster 2 (45.6%) – highly deficient: carbohydrate deficient, lipid deficient, vitamin B9 deficient, vitamin D deficient, vitamin E deficient, calcium deficient, zinc deficient, excess protein, vitamin B1 deficient, iodine deficient, magnesium deficient, fibre deficient, poor lipid quality, cyclist.

Two main clusters emerged (Fig. 1): CHO, lipid and vitamin E deficiency characterised all clusters, in addition to excess protein intake. The first cluster included 54.4% of the sample. This denotes a profile of individuals taking supplements. Individuals in this cluster typically do not exhibit any further deficiency than that mentioned above but do have an excessive vitamin B3 intake. The second cluster included 45.6% of the sample who demonstrated severe dietary deficiencies. Cyclists were more likely to show this profile. This cluster was characterised by deficiencies in vitamin B9, D and B1, in addition to calcium, zinc, iodine, magnesium and fibre deficiencies. Further, lipid quality within this cluster was generally poor.

## Discussion

The main aim of the present study was to assess the dietary intake of Spanish recreational cyclists and triathletes. The main finding was that a large proportion of the present sample consumed an unbalanced diet, characterised by excess protein, and CHO and vitamin E deficiencies. Females and triathletes appear to follow a healthier diet than males and cyclists given that they consumed greater amounts of vegetables, fruits, nuts, wholegrain cereals and olive oil, in addition to consuming less refined cereals, soft drinks and alcoholic beverages including beer and wine. Females and triathletes also had a higher intake of unprocessed food and a lower intake of ultra-processed food than males and cyclists. A high proportion of triathletes who reported regular supplementation, demonstrated an excessive vitamin B3 intake. On the other hand, cyclists tended to belong to a group of individuals characterised by fibre, calcium, zinc, iodine, magnesium, and vitamin B1, B9 and D deficiencies, in addition to poor lipid quality.

The SENC recommends a minimum daily consumption of two servings of vegetables and up to three servings of fruit^21^. Values from the present study are consistent with these recommendations. When compared with the Spanish population we observe that average fruit and vegetable intake corresponds to approximately two servings a day, falling short of the five servings recommended^22^. Spanish SENC guidelines encourage individuals to eat pulses at least two to four times a week. The present study reported consumption of approximately 2.5 servings a week, this being higher than that previously reported for the Spanish population^23^. The present study shows an average consumption of meat and derivatives that corresponds to approximately 1.5 additional servings to the maximum of three recommended by the SENC. These outcomes are similar to those previously published in the Spanish population^23^ which revealed an average daily consumption of 146 g/day. The present study shows a higher intake of fish and seafood than that recommended by the SENC (2–3 portions per week). However, the average intake of 88.9 g/day was similar to that found in the Spanish population^24^. Dietary guidelines for the Spanish population^21^ recommended between 3 to 5 servings of eggs a week. Consumption in the present study was lower than this, whilst also being slightly lower than that previously reported in the Food Consumption Survey^25^ (32 g/day). With regards to olive oil consumption, similar trends have been reported previously by the ANIBES^23^ study, corresponding to 17.5 g/day.

The present study demonstrates different food consumption patterns between men and women. Women presented a higher intake of unprocessed food and a lower intake of ultra-processed food with higher levels of vegetables, fruits, nuts, wholegrain cereals and olive oil, alongside a lower intake of refined cereals, baked goods and pastries, sugar and sweets, and drinks and alcoholic beverages including beer and wine. These outcomes are similar to those reported by Fagerli and Wandel^26^ which showed women to follow a healthier diet than men. A possible explanation for this is that women are often more weight-conscious and believe that it may be advisable to limit carbohydrates and fat consumption. Further, this is the first study to show differences in food consumption patterns between recreational triathletes and cyclists. Triathletes showed a higher intake of unprocessed food and a lower intake of ultra-processed food with greater consumption of fish and seafood, vegetables, fruits, nuts, wholegrain cereals and olive oil, alongside lower consumption of refined cereals, and drinks and alcoholic beverages including beer and wine. A possible explanation for this might be that triathletes have a higher socioeconomic status^16^. Further, cyclists tend to follow “old” nutritional strategies passed through word of mouth, whereas triathletes, taking part in a “new” sport relative to cyclists, do not have this problem. However, this aspect needs to be more deeply studied.

Mean total CHO intake was 246.0 ± 88.1 g/day. This is slightly higher than the 185.4 ± 60 g/day reported in previous studies with Spanish populations^27^. However, findings uncovered by the present study (46.8% ± 7.1), represent values that are still below the lower limit of 50%-60% total energy recommended by the SENC^28^. The American College of Sport Medicine recommends that CHO intake should range from 6 to 10 g/kg of BM for athletes^3^. This is due to the fact that carbohydrate maintains blood glucose levels during exercise and replaces muscle glycogen. In the present study, participants average CHO consumption amounted to 3.5 ± 1.4 g/kg of BM in general and was higher in woman (4.2 ± 1.6 g/kg of BM) than in men (3.4 ± 1.4 g/kg of BM). Another study with non-elite athletes competing in endurance sports showed that between 23 and 54% of athletes did not consume the recommended amounts of CHO^29^.

Total fat intake should constitute more than 20% of total energy intake. Failing to reach this intake could impede correct absorption of lipid-soluble vitamins, whilst failing to provide sufficient essential fatty acids^3^. When engaging in moderate physical activity, 30% of energy from fat intake is recommended. This increases to 35% when engaging in high physical activity^30^. Values reported in the present study met SENC recommendations that total fat intake should account for 30–35% of total energy^31^. Similar results have been reported for the Spanish population with a total fat intake of 78.7 (26.5) g/day in 18–64-year-old adults^27^.

With regards to the quality of fats, in 2010^32^ WHO/FAO recommended a maximum intake of SFA of 10% of total energy due to the association between SFA consumption and cardiovascular disease risk^33^. PUFA consumption should fall within a range of 6–11%, whilst MUFA intake can account for up to 15–20% of energy from total fat intake. Results from the ANIBES study in Spain showed SFA intake to be above recommended levels regardless of age and sexes^27^, with similar trends also emerging across most countries^34^. A healthier SFA intake (10.0 ± 2.5%) was found in the present sample of recreational Spanish athletes. It has been suggested that MUFA induces a protective effect against metabolic syndrome and cardiovascular disease risk factors^35^. Further, PUFA, especially docosahexaenoic acid (DHA) and eicosapentaenoic acid (EPA), has been recommended for its potential benefits for human health^36^. In the present study, MUFA and PUFA intake was within recommended ranges (14.8 ± 3.5% and 6.4 ± 1.8, respectively), although slightly higher percentages were found in women than men. In general, these results are similar to the results reported in the ANIBES study^27^, however, this previous study did not find sex- or age-related differences.

In the present study, overall protein intake was well above current recommendations for the Spanish adult population, which stipulates an upper limit of 15% of total energy^31^, or a total protein intake of about 0.8 g/kg BM. Similar trends were observed by the Spanish National Survey of Dietary Intake^24^ and the European Food Safety Authority in 2012^37^. The present study also showed higher protein intake than that recommended by the American Dietetic Association of Canada and American College of Sports for endurance athletes (1.2 to 1.4 g/kg BM/day)^3^. Protein is a crucial macronutrient for providing substrates for the repair and remodelling of muscle and body proteins^38^. In the present study, males reported the upper limit of this recommendation, whilst females exceeded this by consuming 1.7 ± 0.5 g/kg BM/day. A diet that is high in protein and low in CHO is not only typical in Western countries but typical of professional athletes in many different countries^39,40^. Individuals who consume this type of diet risk suffering metabolic acidosis, which can negatively impact athletic performance^6^.

The intake of all vitamins and minerals exceeded their respective RDA or AI, with the exception of vitamins D and E and, in some cases, calcium. Exercise stresses many of the metabolic pathways where micronutrients are required and exercise training may result in muscle biochemical adaptation that increases micronutrient needs. The most common vitamins and minerals of concern to athletes are calcium and vitamins D, C, E and B complex, alongside iron, magnesium and selenium^41^. Our study showed an excess of all of the above apart from vitamins D and E. Vitamin D is required for adequate calcium absorption and bone health^31^. However, as vitamin D is not only obtained from the diet but can also be obtained through sun exposure, vitamin D inadequacy may be diagnosed by evaluating serum total 25-(OH)-vitamin D. Low vitamin E intake is common within European and US populations and could be the result of the low stability of this vitamin in vegetable oils^42^.

Supplements are commercially available products used to complement the normal diet through the provision of additional vitamins, minerals, amino acids, etc^43^. Supplements are normally used because of the ergogenic effects of the vitamins or minerals they comprise on recovery from exercise and health^10^. However, consumers tend not to use this product for their intended purpose and they are not compatible with normal eating habits^44^. To our knowledge, this is the first research study to examine the prevalence of supplementation in recreational endurance athletes whilst also considering the two different sporting disciplines of cycling and triathlon. The proportion of participants that reported often taking supplements in our study is much lower than that reported in other studies with professional athletes^45,46^. The majority of participants who took supplements opted to take protein, multivitamins, minerals and omega-3. A certain degree of supplementation can be justified. For instance, some women may supplement with iron. Iron deficiency can impair muscle function and limit work capacity, resulting in impaired training adaptations and poorer performance^47^. The present study did not find differences in iron intake between women of different specialities. Both cyclists and triathletes generally met iron requirements. However, other supplements, such as multivitamins, are recommended only when deficiencies are present and only under professional supervision^10^. Nonetheless, a number of previous studies have shown that athletes most frequently consume multivitamins and mineral supplements without supervision, instead using them indiscriminately at their own discretion^48^. Protein supplementation also seems to be inappropriate as many participants already exceeded guideline amounts of protein through their regular diet alone.

The present study revealed a group of participants who tended to be triathletes, supplemented often and consumed an excessive amount of vitamin B3. Excessive vitamin B3 intake could provoke flushing, headaches, light-headedness, itching, nausea and vomiting. In addition, liver injury can occur in rare cases and progress to fulminant hepatic failure^49^. As the indiscriminate use of vitamin and mineral supplements may adversely affect physiological function and impair health^50^, it is recommended to consume high nutrient density food rather than nutritional supplements. Another group, mostly formed of cyclists, was characterised by deficiencies in vitamin B9, D and B1, in addition to calcium, zinc, iodine, magnesium and fibre deficiencies. Further, lipid quality within the cluster was generally poor. Even though a high percentage of the overall sample reported high micronutrient intake in general, we found a cluster of mostly cyclists whose consumption was less than the required amount. Perceptions that supplements could substitute a well-balanced diet may explain these deficiencies.

A limitation of the present study is its cross-sectional design, which inhibits examination of causal relationships, whilst the relatively small number of females could have masked further gender differences. Further, measurement error is an inherent risk of self-report questionnaires. However, the FFQ has previously demonstrated high validity and reliability within similar populations. We used the Willett method^13^ to exclude participants reporting excessively low or high energy intakes. An advantage of this method is that it provides a consistent protocol in cases where the dietary-report instrument employed does not allow for an accurate estimation of energy intake, as is the case with food frequency questionnaires. However, the method still assumes that all values exceeding a given value are inappropriate without considering the activity level of individuals. Height and weight were self-reported as opposed to directly measured due to time, financial resources and manpower constraints. While this method is less accurate than direct measurement, it has demonstrated good agreement and validity in healthy weight populations.

## Conclusions

Spanish recreational athletes follow unbalanced diets which are low in CHO and high in protein, although fat consumption appears to be adequate. The importance of nutritional education for recreational athlete populations should be highlighted in order to optimise CHO and protein intake. Improving dietary strategies may lead to better training adaptations and health. The issue of dietary supplementation misuse can be tackled and so nutritionists should work with teams and athletes to give individualised nutritional advice.
